# The Predisposing Factors of War Psychoneuroses

**Published:** 1918-03

**Authors:** 


### NERVOUS

---

***The Predisposing Factors of War Psychoneuroses.*** By J. M. Wolfsohn, Capt. M. R. C., Abs. by the Author from *The Lancet*, Feb. 2. 1918.

One hundred cases of war psychoneuroses, including shell shock, neurasthenia, hysteria, exhaustion, etc., and one hundred cases of gun-shot and other injuries, causing somatic wounds, were studied by the author, from the family and previous history of the patient.

The family history revealed in the neurosis group, insanity in the family in 34 o/o of cases, epilepsy in 30 o/o, stigmata of degeneration in 10 o/o, while there were none of these factors present in the control group.

Nervousness in the forbears, e. g., history of phobias, insomnia, nerves or hysteria, were present in 64 o/o of the neurotics and in only 15 o/o of the controls.

Excessive use of alcohol in 50 o/o and total abstinence in 30 o/o of the parents or grandparents of the neurotics, whereas 24 o/o and 16 o/o respectively were found in the controls.

A positive family history was found in 74 o/o of the neurotics and in 38 o/o of the controls.

In the personal history, previous to enlistment, all the symptoms mentioned, which include previous nervousness, fears, head injuries, epilepsy, excessive alcohol, excessive tobacco, total abstinence, depression, previous breakdowns, enuresis, excessive religion, were much more commonly found in neurotics than in controls; especially is this true of those who suffered from previous nervousness and timorous disposition. 66 o/o against 12 o/o in these cases. 48 o/o of neurotics were total abstainers, compared with 20 o/o of controls. A tetotaller is usually the offspring of alcoholic forebears, and usually represents the reaction in the other direction.

The neurotic patient is generally moody and asocial, 55 o/o as compared with 8 o/o of controls. Examples, typifying cases of shell shock are given.

76 o/o of the neurotic patients give positive personal history, compared with 30 o/o of controls.

14 o/o of neurotics had relapses and recurrences of their neuroses, there being none in the controls.

The neurosis was acquired in 12 o/o of the former and in 3 o/o of the latter.

The history of present illness with its symptoms shows a most striking comparison, the chief symptoms being charted below :

*Present Illness.*

Percentages of conditions named in (A) Neurosis, (B) Wounded.

| | (A) | (B) | | (A) | (B) |
|---|---|---|---|---|---|
| Service . . . . . . | 12 mos. | 12 mos. | Fears. . . . . . | 76 | 0 |
| Unconsciousness. . | 55 | 24 | Dreams. . . . . | 88 | 50 |
| Dazed. . . . . . . | 84 | 24 | Fatigue . . . . . | 94 | 40 |
| Tremor. . . . . . | 84 | 12 | Headache . . . . | 88 | 36 |
| Poor memory . . . | 88 | 4 | Moody . . . . . | 92 | 0 |
| Poor concentration . | 88 | 4 | Vertigo . . . . . | 74 | 8 |
| Insomnia . . . . | 86 | 0 | Fits. . . . . . . | 10 | 0 |

*Conclusions.*

From this study of 100 cases of war psycho-neuroses and 100 cases of somatic injuries produced in the firing line one can draw the following conclusions : —

Cases of war neurosis are very rarely associated with external or somatic wounds. The vast majority of the psychoneurotic cases studied were among soldiers who had a neuropathic or psychopathic soil. In 74 per cent. of these cases a family history of neurotic or psychotic stigmata, including insanity, epilepsy, alcoholism, and nervousness, was obtained, whilst a previous neuropathic constitution in the patient himself was present in 72 o/o.

A gradual psychic shock from long-continued fear, together with the sudden change from quiet peaceful environment to the extraordinary stress and strain of trench fighting, is the chief predisposing cause of war psychoneurosis in soldiers with neuropathic predisposition. In fact, these factors may be the cause of the neurosis *per se.*

The history of the individual previous to enlistment has an influence on the character and gravity of the symptoms of the neurosis.

Acquired neuroses with their less severe symptoms appeared only after excessive fatigue, with the concussion from the high-explosive detonation acting as the exciting factor.

On the other hand, in the same abnormal environment, and with the same powerful factors obtaining, wounded soldiers do not suffer from war neuroses except in rare instances. In the wounded soldiers studied no neuropathic or psychopathic stigmata occurred in the family history.

Previous neuropathic tendencies were found in 10 o/o of these patients (controls), all of which number presented mild neurasthenic symptoms.

Hysteric manifestations, such as monoplegia in a wounded limb, are occasionally encountered in injured soldiers.

